# Non-Stationary Viscoelastic Modeling of Compression Creep Behavior in Composite Bolted Joints

**DOI:** 10.3390/polym17101382

**Published:** 2025-05-17

**Authors:** Jingwen Yang, Shuai Wang, Hongli Lu, Zhiwei Yuan, Xiaokai Mu, Qingchao Sun, Bo Yuan

**Affiliations:** 1 School of Mechanical Engineering, Dalian University of Technology, Dalian 116023, China; yangjingwen_dlut@163.com (J.Y.); yuanzhiwei1@mail.dlut.edu.cn (Z.Y.); muxiaokai@dlut.edu.cn (X.M.); qingchao@dlut.edu.cn (Q.S.); 2 Beijing Institute of Astronautical Systems Engineering, Beijing 100076, China; 13810095158@163.com (S.W.); 18610040529@163.com (H.L.)

**Keywords:** polymeric matrix composites, modeling, viscoelastic, bolted joints, creep

## Abstract

Fiber-reinforced polymer (FRP) composites are widely utilized in aerospace and shipbuilding due to their outstanding mechanical properties and lightweight nature. During prolonged service, the mechanical performance of composite bolted joints has drawn increasing attention. This study integrates experimental, theoretical, and numerical methods to simulate compressive creep and clarify preload relaxation mechanisms in these joints. A non-stationary Burgers model is proposed to describe the compressive creep behavior of FRP composites and metals, implemented in ABAQUS, which improves fitting accuracy by approximately 10% in *R*^2^ compared to the classical model. Two types of creep tests were conducted to examine the effects of initial load and material type on creep behavior, with model accuracy validated against experimental data. Finite element analysis (FEA) was further employed to assess the impact of localized loading and structural parameters on strain. The results demonstrate that the viscoelastic behavior of materials is the dominant factor contributing to preload relaxation in composite bolted joints. Under localized loading conditions, the maximum creep strain can be reduced by more than 60%, effectively mitigating preload loss. This study provides a robust framework for predicting preload relaxation, offering valuable insights for composite bolted joint design.

## 1. Introduction

Advanced composite materials, represented by fiber-reinforced polymers (FRPs), are characterized by high specific modulus and high specific strength, making them indispensable in modern industries. They are ideally suited to applications that demand high strength and stiffness, reduced weight, and exceptional fatigue resistance [1,2,3]. These advantages significantly enhance energy efficiency, as in the transportation industry, and weight reduction directly impacts fuel consumption and emissions [4,5]. For instance, the two most recent long-range aircraft, the Boeing B787 Dreamliner and Airbus A350 XWB incorporate over 50% FRPs in the airframe, including large fuselages, wings, tail sections, and other primary aerostructures [6,7].

In most industrial applications, FRPs require joining with metal components to form complete structures while maintaining adequate stiffness and strength [8]. Fiber-reinforced orthotropic polymers and metals possess distinct material properties and manufacturing processes, making it challenging to establish an effective and reliable fastening between them [9]. As a result, design approaches that are traditionally strength-based may transition to being driven by stiffness considerations [10]. The most commonly used jointing methods are bolted joining, adhesive bonding, and hybrid mechanically fastened/adhesively bonded joints [11,12]. Bolted joining offers the advantage of ease of assembly/disassembly, high strength, and dependable connection performance, making it the dominant method for assembling composite structures [13]. The joining technology in composite structures represents a critical challenge, with 60–85 percent of failures occurring at the fastening joints, in contrast to metal structures [14]. Extensive studies have been conducted on various aspects, including stress distribution [15], static and dynamic strength, as well as the associated failure mechanisms [16], and novel anchorage systems [17], all of which contribute to a more comprehensive understanding of the mechanical performance of composite bolted joints.

The intricate stress distribution near joints can result in multiple failure modes in composite structures [18], making the required preload a critical factor in ensuring the mechanical performance of bolted joints [19]. Consequently, the evolution of bolt preload is of paramount importance in the design and evaluation of composite structures. A polymer matrix of composite materials typically exhibits viscoelastic behaviors at ambient temperature [20,21]. In a strict sense, loosening refers to the rotational process of the internal and external threads in the direction opposite to the tightening direction, which results in a reduction in the bolt preload [22]. However, the preload in threaded fasteners may decrease even without a reverse rotation between the internal and external threads [23]. This process is typically divided into the following stages: (1) short-term relaxation resulting from surface strain hardening; (2) localized plastic deformation; and (3) long-term preload relaxation caused by the creep of clamped joints. FRP composite laminates are the primary cause of preload relaxation, with their properties predominantly determined by the creep behavior of the resin matrix [24].

Caccese et al. [25] conducted extensive experimental studies to investigate the factors affecting the preload relaxation in hybrid composite/metal joints. The results indicated that temperature, humidity, and stress distribution conditions all play significant roles in influencing preload relaxation. Finck et al. [26] studied the preload relaxation behavior of thermoset carbon fiber sheet molding compounds in screwed connections at a 120 °C ambient temperature. The results showed that the creep laws of isotropic materials can effectively evaluate the loss of preload due to creep. The research also demonstrated that using conical spring washers can effectively limit preload relaxation. Liu et al. [27] investigated the long-term preload relaxation of space connection and separation devices through experimental methods, revealing that the relaxation behavior exhibits a two-stage feature. In the first stage, the relaxation is attributed to the deformation and gap adjustment of the components, while the second stage is mainly induced by material creep. Wang et al. [28] investigated the effect of epoxy adhesive creep under varying temperatures on the performance of CFRP plate strengthened metallic beams based on a linear viscoelastic model. The results showed that warm service temperatures significantly amplify the linear viscoelastic creep of the adhesive, thereby increasing the risk of long-term slip and bond failure in FRP-strengthened structures. Lal et al. [29] conducted a comprehensive experimental investigation into the synergistic effects of cyclic and sustained bending loads coupled with water immersion on the interfacial shear strength of CFC/GFRP hybrid rods, revealing the evolution mechanism of the hybrid rods under hygrothermo-mechanical coupling conditions.

Early studies on creep phenomena were primarily experimental, with various semi-empirical creep theories being established based on experimental data by fitting creep curves. These theories include the time-hardening theory [30], strain-hardening theory [31], and plastic hysteresis theory, among others. Early research on metallic joints revealed that bolt preload decreases due to the creep of surface coatings. Yang et al. [32] conducted detailed theoretical and experimental studies on this subject. According to their research, preload relaxation is governed by multiple factors, with the coating’s creep strain following a power-law expression. This finding demonstrates that coating creep persists over an extended duration and significantly contributes to preload loss. Shivakumar [33] proposed an empirical equation for the degradation of bolt preload under transient temperature-humidity conditions, which was used to calculate the long-term relaxation behavior of graphite/epoxy composite joints under varying temperature and humidity conditions. The results indicated that as the environmental temperature increases, the preload relaxation also becomes more significant. Horn et al. [20] conducted short-term preload relaxation tests for two types of thermoplastic composite joints at room temperature and high temperatures throughout 700 to 1400 h. By combining the preload relaxation test data with Equation (2), they predicted the relaxation of preload after 100,000 h. The results showed that the preload relaxation in composite joints at high temperatures was considerably greater than that at room temperature. Bouzid et al. [34,35] investigated leakage issues in high-temperature flange connection structures during service and used a logarithmic function to fit the creep strain of gaskets. Kallmeyer et al. [36] investigated the localized creep response of composite joints under both ambient and elevated temperatures. The study also proposed an empirical expression for the relationship between creep, time, and contact stress based on the time-hardening theory. Nechache et al. [37] developed a model for the analysis of preload relaxation that considers the creep behavior of the bolt, flange, and gasket. Scattina et al. [38] experimentally investigated the creep relaxation characteristics of hybrid composite-to-metal joints, examining the effects of various factors such as temperature, stress, and surface roughness on creep behavior. This work also compared the fitting performance of linear, logarithmic, and power-law equations. The results indicated that each of the three fitting equations had its advantages and disadvantages under different loading conditions.

The classical semi-empirical creep theories derive analytical expressions for creep strain through the use of experimental data and empirical observations. Although these methods are straightforward and effective, they lack general applicability and have limited capacity to elucidate the underlying mechanisms governing creep phenomena. With the continuous development of viscoelastic constitutive theories and numerical calculation methods, mechanical models such as viscoelasticity have been widely applied in simulating creep behavior. Classical mechanical models include the Maxwell model, Kelvin model, Zener model, and Burgers model [39]. The Maxwell model can be represented by a viscosity element and an elastomer arranged in series, which is commonly used to describe the stress relaxation behavior of linear polymers. The Voigt–Kelvin model can be represented by a damper and an elastic spring arranged in parallel, which is primarily used to describe the creep behavior of cross-linked polymers. The Zener model, also known as the standard linear solid model, is typically used to describe the creep behavior of ideal cross-linked polymers. The Burgers model is a widely accepted model for the time-dependent behaviors of ideal linear polymers [40]. Abboud et al. [41] conducted research on the creep relaxation behavior of gaskets under compression step-load conditions at environmental temperatures for bolted joints based on the Zener model. The research investigated the effects of gasket material, geometric parameters, and load duration on creep behavior. The results indicated that the Zener model can accurately predict the loss of clamping load due to gasket creep in bolted flange joints. Wang et al. [42] proposed a micromechanical model based on the Burgers model for predicting preload relaxation in composite joints, considering the fiber volume fraction in composites. Hu et al. [43] utilized the Burgers model to analyze the preload relaxation mechanism under thermal loading for interference/fit composite joints. The findings indicated that an increase in interference or a decrease in temperature both contribute to alleviating the preload relaxation phenomenon. Li et al. [44] proposed a physical model, based on the Burgers model, to describe the relationship between creep displacement of rough surfaces in contact and preload relaxation. Lv et al. [45,46] presented an integrated experimental/theoretical/numerical approach, considering the tensile/compressive asymmetry of composites, which was implemented using FEA in ABAQUS software. Xie et al. [47] established a creep model for composite materials considering rough contact surfaces and predicted the preload relaxation behavior of composite joints.

However, although extensive research has been conducted on preload relaxation in composite bolted joints, traditional viscoelastic models—such as the classical Burgers and Zener models—exhibit significant limitations in accurately describing the time-dependent creep behavior of materials and metals because they fail to account for the time-varying characteristics of these materials. Moreover, existing studies have primarily focused on fitting preload degradation curves, with few investigating the underlying mechanisms of material creep; as a result, the creep-induced preload relaxation mechanism in composite bolted joints remains poorly understood.

This paper presents an experimental/theoretical/numerical approach to simulate the compression creep behavior of composite materials, metals, and their contact pairs in bolted joints. A non-stationary Burgers model was proposed to characterize the creep behavior of resin, FRP composites, and metal, implemented in ABAQUS 2021 (Dassault Systèmes Simulia Corp., Providence, RI, USA) using UMAT and SDVINI subroutines. Two types of creep tests (material and contact pair tests) were conducted to examine the effects of initial load and material type on creep behavior, with model accuracy validated against experimental data. Numerical methods were used to investigate the impact of localized loading and structural parameters on strain, aiming to clarify the mechanisms and provide insights for engineering applications.

## 2. Materials and Methods

### 2.1. Materials

**Figure 1:**
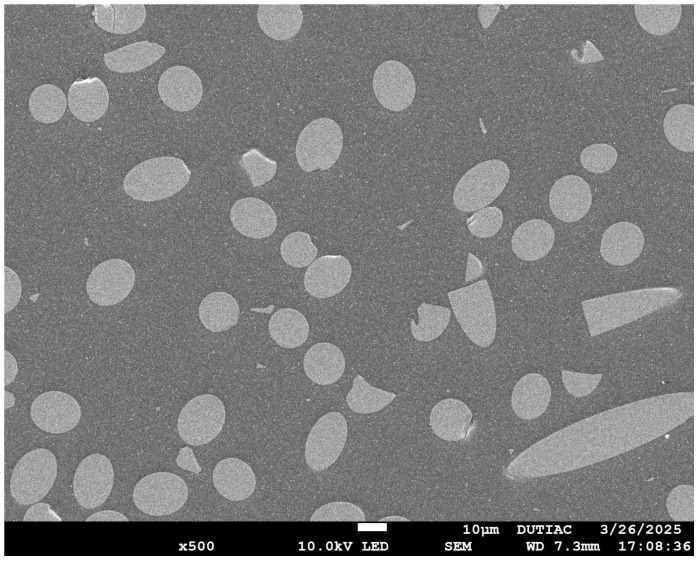
The SEM image of FR-PEEK. (JSM-7610 Plus (JEOL Ltd., Tokyo, Japan)).

This study investigates the creep behavior of materials in bolted connections, focusing on polyetheretherketone (PEEK), fiber-reinforced polyetheretherketone (FR-PEEK), and 2A14 aluminum alloy (2A14) for the joints and 13-8 Mo stainless steel (13-8Mo) as the bolt material (treated as purely elastic). FR-PEEK is a short glass-fiber-reinforced composite with a fiber volume fraction of 30% and a fiber width of approximately 15 μm. The SEM image of the material is shown in Figure 1. PEEK is a high-performance thermoplastic widely used in aerospace, automotive, and electronics due to its excellent mechanical and thermal properties. When reinforced with chopped glass fibers, PEEK becomes significantly stronger and stiffer, making it suitable for structural applications like bolted joints in composite assemblies. Materials were selected for analysis in this study considering the widespread use and stable mechanical properties of chopped-glass-fiber-reinforced PEEK composites. And all experiments were conducted at ambient temperature. The tests include homogeneous contact pairs (PEEK/PEEK, FR-PEEK/FR-PEEK, and 2A14/2A14) and heterogeneous contact pairs (FR-PEEK/2A14 and FR-PEEK/13-8Mo). The properties of the material are reported in Table 1.

### 2.2. Non-Stationary Burgers Model

Considering long-term creep behavior, the mechanical performance of resin matrices and composites degrades over time, necessitating an appropriate constitutive model to describe the compression creep behavior of joints. In practical applications, the viscoelasticity of polymers changes with time. The deformation of FRP composites under compressive loading does not grow indefinitely nor remain completely stable. To address the limitations of classical viscoelastic models in characterizing time-dependent behavior, this paper introduces a non-stationary Burgers model for accurate characterization of material viscoelasticity while maintaining engineering simplicity.

The classical Burgers model is composed of a Maxwell model and a Kelvin model arranged in series. According to the principle of serial connection, the total strain in the Burgers model can be expressed as [52]:

(1) $$\varepsilon \left(t\right)=\varepsilon_{e}+\varepsilon_{v}\left(t\right)+\varepsilon_{k}\left(t\right)$$

where $\varepsilon \left(t\right)$, $\varepsilon_{e}$, $\varepsilon_{v}\left(t\right)$, and $\varepsilon_{k}\left(t\right)$ represent the strain of the Burgers model, the elastomer, the viscosity element, and the Kelvin model, respectively, with units of %. The creep equation under the applied load is obtained as follows:

(2) $$\varepsilon \left(t\right)=\frac{\sigma }{E_{0}}+\frac{\sigma }{\eta_{2}}t+\frac{\sigma }{E_{1}}\left[1-\exp\left(-E_{1}t/\eta_{1}\right)\right]$$

where σ is the stress, MPa. $E_{0}$ and $E_{1}$ are the elastic modulus of the elastomers, MPa. $\eta_{1}$ and $\eta_{2}$ are the viscosity coefficient of the viscosity elements, MPa·h.

**Figure 2:**
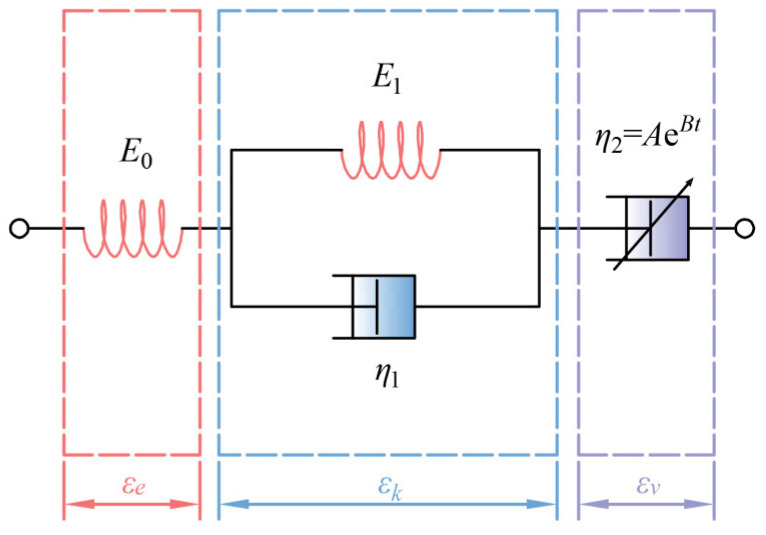
The non-stationary Burgers model.

In the classical Burgers model, if the duration of loading is sufficiently long, the strain is predicted to increase indefinitely in a linear fashion, which deviates from actual behavior. In practical engineering scenarios, the creep rate of polymers decreases over time due to factors such as material aging. Therefore, a nonlinear correction is applied to the viscosity coefficient $\eta_{2}$ in the classical Burgers model. The modified mathematical expression for the viscosity element is given as follows:

(3) $$\eta_{2}=A\exp\left(\mathrm{Bt}\right)$$

where *A* is the initial damping, and *B* represents the aging coefficient. The non-stationary Burgers model, incorporating a nonlinear time-dependent viscosity element, established in this study, is shown in Figure 2. Under the specified loading conditions, the creep equation for the non-stationary Burgers model is given as:

(4) $$\varepsilon \left(t\right)=\frac{\sigma }{E_{0}}+\frac{\sigma }{\mathrm{AB}}\left[1-\exp\left(-\mathrm{Bt}\right)\right]+\frac{\sigma }{E_{1}}\left[1-\exp\left(-E_{1}t/\eta_{1}\right)\right]$$

where $\varepsilon_{e}$ represents the strain that is independent of time, which corresponds to the instantaneous elastic strain occurring under the applied load; $\varepsilon_{c}\left(t\right)$ represents the time-dependent strain, which is the strain under constant load. As the parameter *B* approaches zero, the nonlinear viscosity element may degenerate to a linear one, in which case the non-stationary Burgers model reverts to its classical form. When the parameter *B* is greater than or equal to 1, the viscosity element becomes sufficiently stiff, causing the non-stationary Burgers model to degenerate into the Zener model.

**Figure 3:**
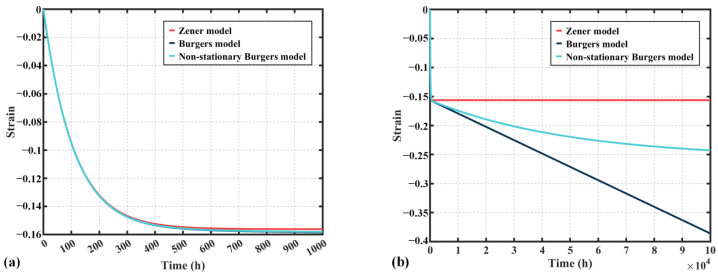
Comparison of the three models: (**a**) 1000 h, (**b**) 100,000 h.

Figure 3 illustrates the compression creep strain curves over 100,000 h for the Zener model, Burgers model, and the non-stationary Burgers model proposed in this study, under identical material parameters. It can be observed that, after an extended period, the creep strain in the Zener model levels off, whereas the Burgers model exhibits a linearly increasing creep strain. In contrast, the non-stationary Burgers model proposed in this work shows a continuous decrease in the relaxation rate until it stabilizes, at which point the creep strain remains relatively constant.

### 2.3. Numerical Implementation

The non-stationary Burgers model was further developed through the UMAT subroutine in ABAQUS software. Initially, the expressions derived for the one-dimensional stress state need to be extended to a three-dimensional stress state. According to the theory of continuum mechanics, the stress $\sigma_{\mathrm{ij}}$ at any given point can be represented by the spherical stress tensor $\sigma_{m}\delta_{\mathrm{ij}}$ and the deviatoric stress tensor $s_{\mathrm{ij}}$, while the strain $\varepsilon_{\mathrm{ij}}$ at any given point can be described by the spherical strain tensor $\delta_{\mathrm{ij}}\varepsilon_{kk}$ and the deviatoric strain tensor $e_{\mathrm{ij}}$:

(5) $$\left\{\begin{array}{l} \sigma_{\mathrm{ij}}=\delta_{\mathrm{ij}}\sigma_{kk}+s_{\mathrm{ij}}\\ \varepsilon_{\mathrm{ij}}=\delta_{\mathrm{ij}}\varepsilon_{kk}+e_{\mathrm{ij}} \end{array}\right.$$

the constitutive equation for the elastic element in three-dimensional form is:

(6) $$\varepsilon_{\mathrm{ij}}=\frac{1}{3Κ}\delta_{\mathrm{ij}}\sigma_{\mathrm{kk}}+\frac{1}{2G}s_{\mathrm{ij}}$$

FRP composites do not exhibit a distinct yield limit, and thus it can be assumed that the yield stress is approximately zero [43]. In this case, the creep strain can be analyzed using plastic flow theory. Based on the Von Mises theory, the equivalent stress $\bar{\sigma }$ is given by:

(7) $$\bar{\sigma }={\left(\frac{3}{2}s_{\mathrm{ij}}s_{\mathrm{ij}}\right)}^{1/2}=\sqrt{3J_{2}}$$

where *J*_2_ is the second invariant of the deviatoric stress tensor, expressed as:

(8) $$J_{2}=\frac{1}{2}\left(s_{1}^{2}+s_{2}^{2}+s_{3}^{2}\right)=-\left(s_{1}s_{2}+s_{2}s_{3}+s_{3}s_{1}\right)$$

based on the orthogonal law of plastic mechanics:

(9) $${\dot{\varepsilon }}_{\mathrm{ij}}^{c}=\lambda \left(\frac{\partial f}{\partial \sigma_{\mathrm{ij}}}\right)=\lambda s_{\mathrm{ij}}$$

The relationship between equivalent strain and equivalent stress is given by:

(10) $$\lambda =\frac{3{\overset{\dot{¯}}{\varepsilon }}^{c}}{2\bar{\sigma }}$$

The combination of Equations (11) and (12) leads to:

(11) $${\dot{\varepsilon }}_{\mathrm{ij}}^{c}=\frac{3{\overset{\dot{¯}}{\varepsilon }}^{c}s_{\mathrm{ij}}}{2\bar{\sigma }}$$

The creep strain increment at each step in the finite element analysis can be written as:

(12) Δεijcn+1=∫tt+Δt32ε¯˙cn+1tσ¯n+1tsijn+1tdτ

where *D_e_* represents the elastic tensor. The iterative solution format for the constitutive relation can be expressed using the implicit central difference method:

(13) Δεkcn+1=C⋅βkn+1t+ΘΔtΔtσ¯kn+1t+ΘΔt k=0,1,2,⋯

(14) ε¯˙kcn+1t+ΘΔt=1−Θε¯˙ct+Θ⋅ε¯˙kcn+1t+Δt

(15) skn+1t+ΘΔt=1−Θskn+1t+Θ⋅skn+1t+Δt

where $C=\left[I-(1/3)mm^{T}\right]$, $m^{T}=\left[\begin{array}{cccccc} 1 & 1 & 1 & 0 & 0 & 0 \end{array}\right]$. The algorithm can ensure stability when θ≥1/2. And the equation for solving σk+1n+1t+Δt is given by:

(16) σk+1n+1t+Δt=σt+Δσkn+1

The UMAT subroutine for the non-stationary Burgers model can be developed with the above iterative format. In the absence of plastic strain and thermal strain, only creep strain and initial strain are considered. The Jacobian matrix based on the constant stiffness iteration can be expressed as:

(17) A=λ+2Gλλ   λλ+2Gλ   λλλ+2G      G      G      G

Based on the numerical framework outlined above, the UMAT subroutine for the non-stationary Burgers model can be implemented.

### 2.4. Finite Element Model

**Figure 4:**
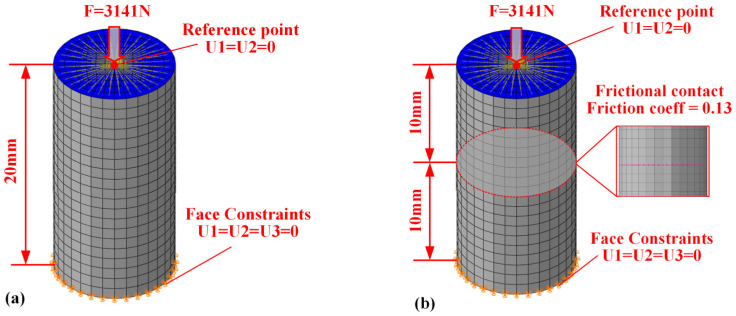
Finite element model (mesh details, boundary, and load conditions) for material specimen.

A finite element model of the specimen was created, with dimensions fully consistent with the actual experimental specimen. The model was discretized using fully integrated C3D8 elements, resulting in a final model with 3190 nodes and 2560 elements. Boundary conditions were applied with axial loads at one end at the reference point, and the load was maintained as in the experiment. To prevent rigid body motion, the U1 and U2 degrees of freedom at the reference point were constrained, and the opposite end face was fixed with constraints on the U1, U2, and U3 degrees of freedom, as depicted in Figure 4a. Simulation of the creep behavior of contact pairs was conducted by establishing a finite element model (Figure 4b) of the contact pair, with dimensions fully consistent with the actual experimental specimen. The model was discretized using fully integrated elements (C3D8), resulting in a final model consisting of 3190 nodes and 2560 elements. Boundary conditions were applied with axial loading at one end at the reference point, with the loading conditions matching those of the creep experiment. To prevent rigid body motion, the degrees of freedom (U1 and U2) at the reference point were constrained, while the other end face was fixed with constraints on all three degrees of freedom (U1, U2, and U3). A penalty formulation is employed, and a friction coefficient of 0.13—drawn from experimental studies on polymer/metal interfaces—is assigned. Hard contact is used to prevent penetration, with a small overclosure tolerance of 0.01 mm to stabilize convergence. Because our focus is on validating the constitutive behavior of the bulk materials, we assume ideally smooth contact surfaces; the effects of real surface roughness and micro-asperity creep will be addressed in future work.

## 3. Experimental Tests

### 3.1. Problem Statement

The composite bolted joint is a system comprising various components, such as fasteners and connected components, with contact surfaces, including those between the fastener and component, between the connected components, and between threads.

The following assumptions are made: (1) The bolt and nut are assumed to remain within the elastic range, as preload in composite joints is typically small and the materials behave elastically. (2) Preload application occurs quickly, with materials assumed to be elastic during preload application and only exhibiting viscoelastic behavior afterward. (3) The effect of thread contact on preload is neglected.

**Figure 5:**
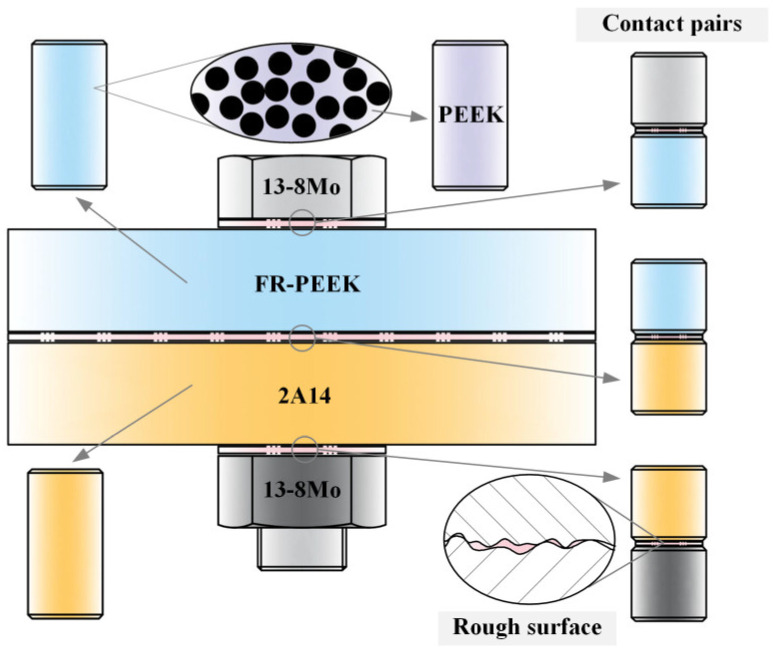
Test specimen design scheme.

Based on these assumptions, as shown in Figure 5, the research focuses on two areas: (1) Design of creep test specimens to investigate the behavior of metals, resin matrices, and fiber-reinforced composites. (2) Design of contact pairs specimens to study the creep behavior of homogeneous/heterogeneous contact pairs, using a combination of simulations and experiments.

### 3.2. Compression Creep Testing Setup

**Figure 6:**
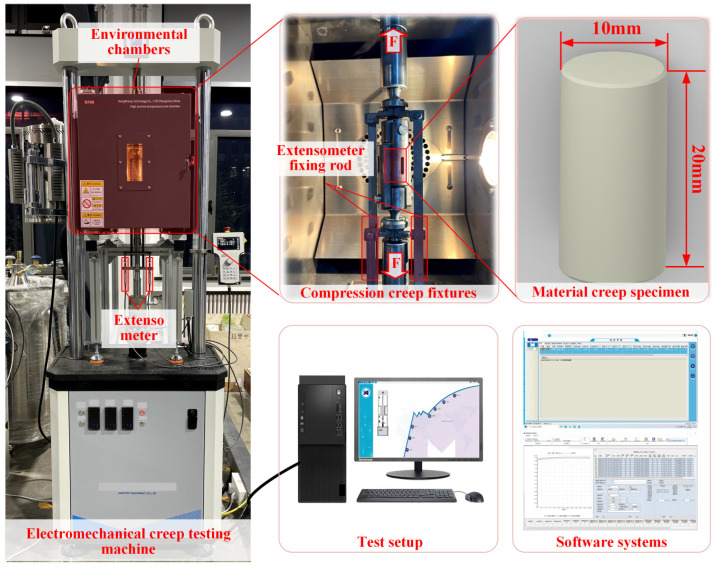
Compression creep test set up.

**Figure 7:**
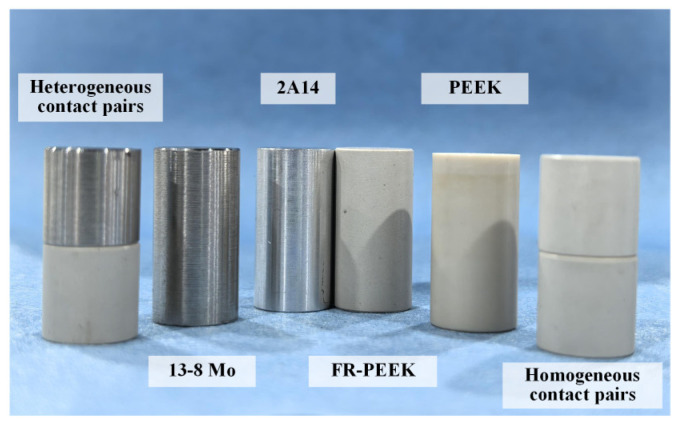
Specimens for the compression creep tests.

Figure 6 shows the experimental setup for compression creep tests. An RDL-30 electronic creep testing machine outfitted with an environmental chamber and high-temperature furnace was used to conduct tests over a temperature range from −180 °C to 1100 °C. A specialized fixture converted tensile load into compressive load, while an extensometer measured real-time creep strain. As shown in Figure 7, cylindrical specimens were designed with the following specifications: material specimens (height 20 mm) for material testing and contact pair specimens (height 10 mm, total thickness 20 mm). To ensure uniform loading, the parallelism of the specimen ends was controlled within 0.01 mm, and the relative strain deviation was maintained within 10% using extensometers at both ends to ensure data reliability and repeatability.

The creep test was conducted with a time control of 48 h, and the test procedure was set to a constant load creep mode, with a load of 40 MPa applied to the specimen. The creep test procedure followed the guidelines of ASTM D2990, with relevant modifications made to adapt to the creep test of the contact pairs [53].

## 4. Results and Discussion

### 4.1. Parameter Determination and Model Validation

The elastic modulus *E*_0_ of the elastomers can be directly identified from the slope of the loading test curve under uniaxial loading conditions, as shown in the following equation:

(18) $$E_{0}=\sigma /\varepsilon_{t=0}$$

where σ is the stress, MPa. $E_{0}$ and $E_{1}$ are the elastic modulus of the elastomers, MPa. $\eta_{1}$ and $\eta_{2}$ are the viscosity coefficient of the viscosity elements, MPa·h.

The remaining four time-dependent parameters $E_{1}$, $\eta_{1}$, A, and B need to be identified. In this study, the nonlinear least squares method is employed to fit the parameters of the non-stationary Burgers model. Specifically, the lsqcurvefit function in MATLAB R2021b (The MathWorks, Inc., Natick, MA, USA) is used, which optimizes the model parameters by minimizing the squared error between the experimental strain data and the model predictions. The results in Table 2 indicate that, for the three materials—PEEK, FR-PEEK, and 2A14—the non-stationary Burgers model demonstrates a highly accurate fit. Compared to the classical Burgers model, the goodness of fit $R^{2}$ has been significantly improved, highlighting the considerable superiority of the non-stationary Burgers model in accurately describing the creep behavior of these materials.

To verify the convergence and accuracy of the non-stationary Burgers model implemented in the UMAT subroutine developed in this work, simulations for the materials mentioned above were conducted and compared with experimental results to assess the subroutine’s accuracy.

**Figure 8:**
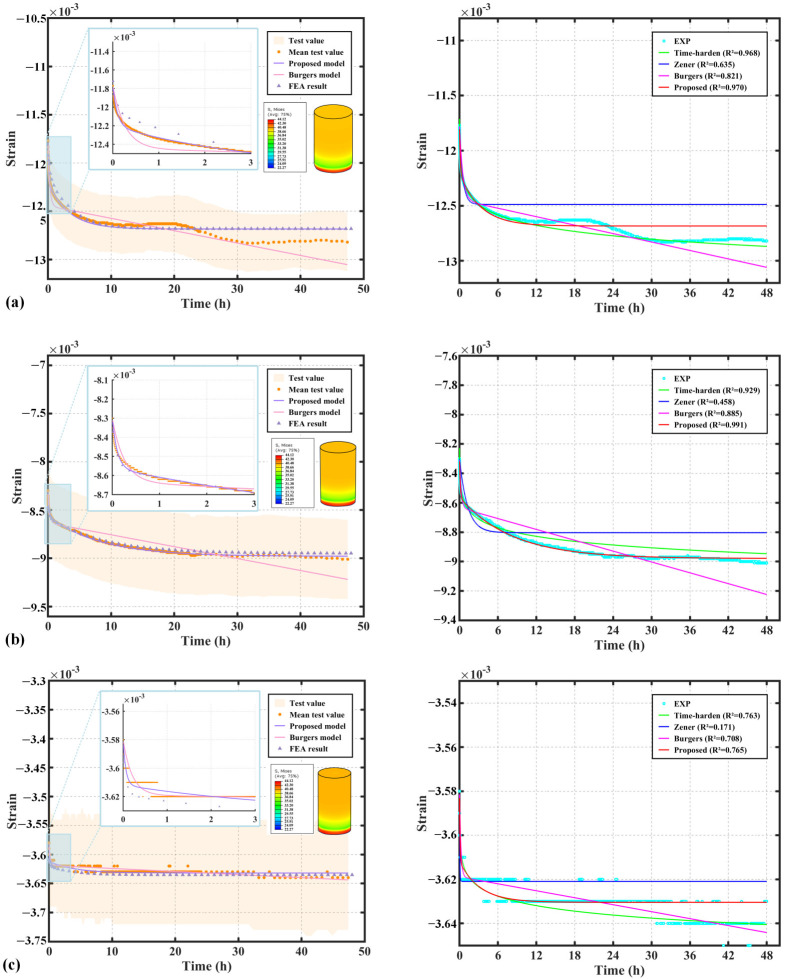
Fitting and simulation results of material compression creep strain curves using viscoelastic models: (**a**) PEEK, (**b**) FR-PEEK, (**c**) 2A14.

Figure 8 presents the fitting curves and simulation results of the proposed non-stationary Burgers model compared with test data, alongside results from the time hardening model, Zener model, and classical Burgers model. As shown in Figure 8a, the non-stationary Burgers model aligns closely with the experimental data for PEEK during the first three hours. After this period, fluctuations in the experimental data lead to increasing deviations in the classical model, which is constrained by its linear viscosity element. In contrast, the non-stationary model remains robust against these fluctuations. Among the four models, the non-stationary Burgers model demonstrates the best overall fitting performance, followed closely by the time hardening model, while the Zener and classical Burgers models exhibit relatively poor agreement.

Figure 8 presents the fitting curves and simulation results of the classical and non-stationary Burgers models for the test data. In Figure 8a, the non-stationary Burgers model matches the experimental data for PEEK excellently within the first three hours, significantly outperforming the classical Burgers model. After three hours, fluctuations in the experimental data cause the classical model, constrained by the linear viscosity element, to deviate, whereas the non-stationary model remains robust against these fluctuations.

Figure 8b illustrates that the proposed model provides a good fit to the creep data of FR-PEEK. The time hardening model also performs well, providing relatively accurate predictions. In contrast, the classical Burgers model, limited by its linear viscosity element, exhibits significant deviations from the experimental curve in the later stages. The Zener model delivers the poorest fitting performance among the four models.

Figure 8c presents the fitting results for 2A14. The material exhibits a rapid initial creep within the first 0.1 h, followed by a “staircase-like” decline. Additionally, the relatively small magnitude of creep strain in 2A14 leads to noticeable data fluctuations, reducing the fitting accuracy of all models. Nevertheless, the proposed model still demonstrates the highest goodness of fit among the compared approaches.

**Figure 9:**
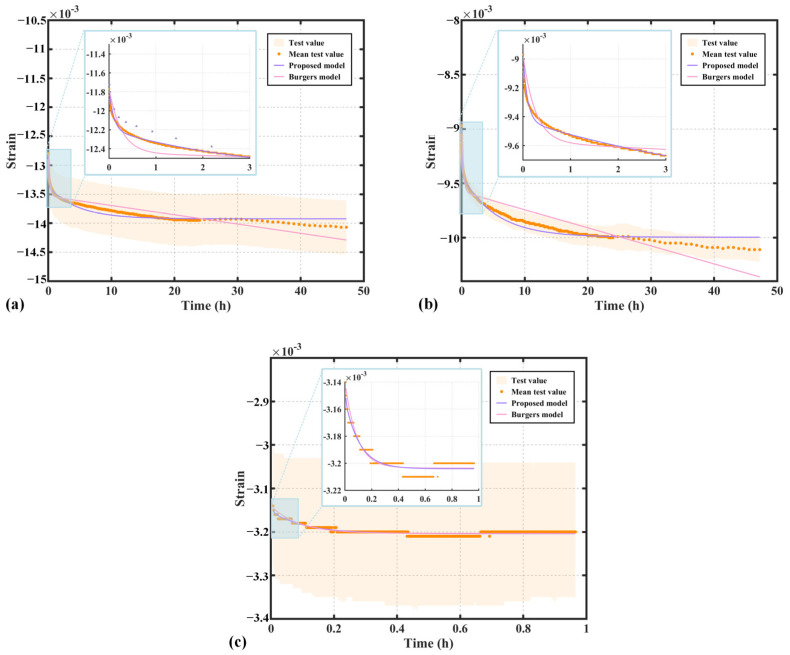
Fitting and simulation results of contact pairs compression creep strain curves using viscoelastic models: (**a**) PEEK, (**b**) FR-PEEK, (**c**) 2A14.

Figure 9 and Table 3 present the fitting results for the compression creep data of the PEEK, FR-PEEK, and 2A14 contact pairs, along with the fitted material parameters. Clearly, similar to the material test results, the non-stationary Burgers model significantly improves the goodness of fit (R^2^) for PEEK and FR-PEEK compared to the classical Burgers model. This demonstrates that the proposed model can satisfactorily simulate the compression creep behavior of contact pairs. Notably, the creep curves of the contact pairs are highly sensitive to the initial preload applied during testing, a relationship that will be discussed in detail subsequently.

In conclusion, the non-stationary Burgers model proposed in this study outperforms the classical Burgers model in fitting the compression creep curves of polymers and their composites. Additionally, it shows lower sensitivity to fluctuations in experimental data and exhibits greater robustness in fitting the strain curve. The behavior obtained through numerical simulations aligns closely with experimental results, confirming the accuracy of the model’s secondary development. This model provides more precise simulation results and holds significant value for engineering applications.

### 4.2. Creep of Contact Pairs

**Figure 10:**
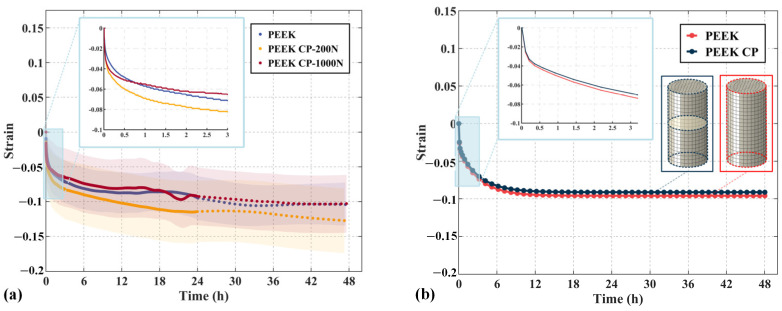
Compression creep behavior of PEEK contact pairs (CPs) under different initial loads: (**a**) experimental results, (**b**) simulation results.

Figure 10 compares the experimental and simulation results for PEEK contact pairs under controlled initial loads. As shown in Figure 10a, at an initial load of 200 N (5% of the test load), the contact pair exhibits a strain of 0.127% over 48 h, which is 22% higher than that of the material specimen. The strain increases rapidly within the first 0.1 h, after which it aligns with the material’s behavior, with both curves running parallel. At an initial load of 1000 N (30% of the test load), the contact pair strain is 0.101%, essentially matching the result of the material specimen. Figure 10b displays the numerical simulation results for the material and contact pair specimens, with simulation errors of 9.5% and 9.9% relative to the experimental data, respectively. This indicates that the simulated compressive creep behavior of the contact pair closely matches the experimental results under high initial loads, whereas a discrepancy of 28.3% is observed under low-preload conditions.

The experimental findings indicate that the contact region diminishes the structure’s resistance to creep, which is likely the primary cause of short-term bolt preload relaxation. Therefore, in engineering applications, it is crucial to consider the creep characteristics of the contact area, and applying preload prior to full tightening can help mitigate preload relaxation.

**Figure 11:**
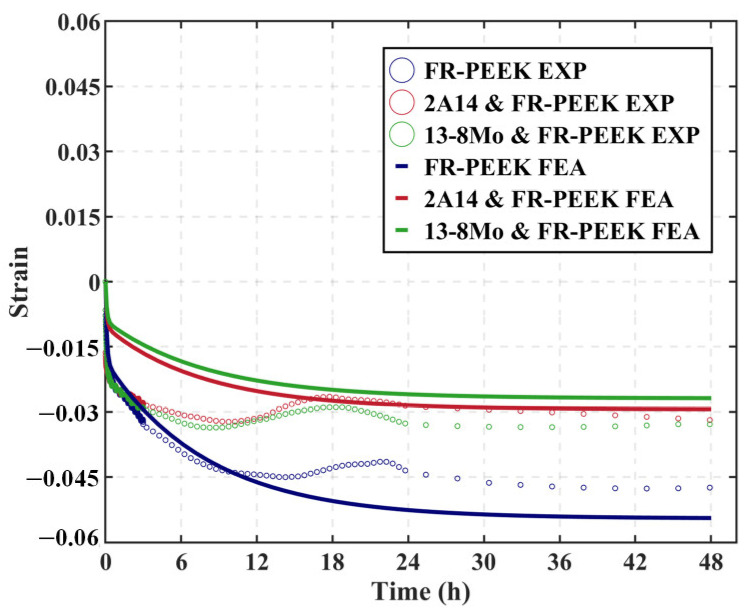
Compressive creep behavior of heterogeneous contact pairs.

Figure 11 compares the creep curves and corresponding finite element analysis results for three heterogeneous contact pair specimens—FR-PEEK/2A14, FR-PEEK/FR-PEEK, and FR-PEEK/13-8Mo—under a preload of 1000 N and identical surface roughness. The experimental creep strains are 0.050% for the FR-PEEK/FR-PEEK pair, 0.034% for FR-PEEK/2A14, and 0.031% for FR-PEEK/13-8Mo. The finite element analysis predicts strains of 0.054%, 0.030%, and 0.027%, respectively, corresponding to errors of 8%, 12%, and 13% relative to the experimental data. These results demonstrate good agreement between simulation and experiment, thereby validating the proposed model and numerical methodology.

Moreover, it is evident that under high-preload conditions the creep behavior of the contact pairs is predominantly determined by the intrinsic material creep, with little influence from the contact interfaces. Consequently, the FR-PEEK contact pair exhibits the highest strain. Considering that the creep effects in 2A14 and 13-8Mo are negligible at ambient temperature—allowing them to be approximated as elastic—the creep strain in the FR-PEEK/2A14 and FR-PEEK/13-8Mo contact pairs is primarily attributed to FR-PEEK, with minimal contributions from the metallic components.

**Figure 12:**
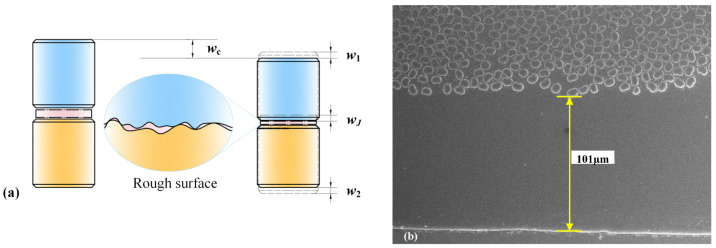
Schematic diagram of microscopic rough surface contact: (**a**) schematic diagram of rough surface, (**b**) surface morphology of the composite material under SEM.

Figure 12 shows the contact pair specimen used in the experiment. Under the condition that the surface quality of the contact surfaces between the test specimen and the fixture is maintained, the creep strain in these two parts of the contact can be neglected. Therefore, the total strain of the contact pair is composed of three parts: the strain of the two materials and the strain between the contact surface, which can be expressed by the following formula:

(19) $$w_{C}=w_{1}+w_{2}+w_{J}$$

where *w*_1_, *w*_2_, and *w_J_* represent the displacements of the contact pair, contact surface, and material 1 and material 2, respectively. Under high initial preload, surface asperities become markedly flattened, yielding negligible interfacial compliance (*w_J_* ≈ 0) and causing the contact pair’s compressive creep response to mirror that of the bulk material. Conversely, at lower preload levels, residual surface roughness permits pronounced time-dependent viscoelastic deformation of the asperities, resulting in significantly greater creep strain in the contact pair than in the material alone. This leads to a substantially larger creep strain in the contact pair compared to that observed in the material alone. The surface morphology of the composite material was observed using scanning electron microscopy (SEM), as shown in Figure 12b. The surface is primarily composed of a resin matrix layer, indicating that the contact interface between the composite and metal is dominated by the resin phase. Hence, under compressive loading, the time-dependent creep behavior at the composite/metal interface is predominantly governed by the viscoelastic deformation of the resin matrix.

**Figure 13:**
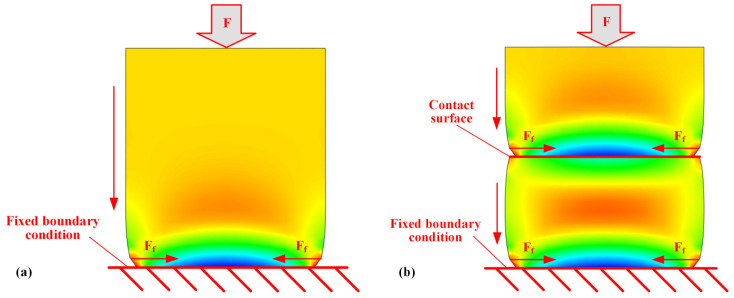
Comparison of the stress fields of material specimens and contact pair (deformation magnified by 30 times): (**a**) material specimen, (**b**) contact pair.

As shown in Figure 13, the stress field distribution during compression creep is illustrated. When the specimen is subjected to compressive load, the bottom tooling primarily constrains it, resulting in a relatively uniform stress field distribution throughout the rest of the specimen (Figure 13a). In contrast, during the compression of the contact pair, the constraints at the contact region, according to the generalized Hooke’s law, partially inhibit the axial compression creep strain (Figure 13b). As a result, the strain of the contact pair, derived from the simulation, is found to be smaller than that of the material itself.

### 4.3. Localized Loading

**Figure 14:**
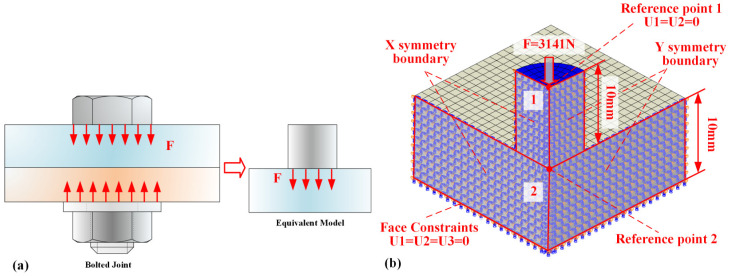
Finite element model for contact pair under localized loading: (**a**) simplified model of the bolt joint, (**b**) finite element model.

Experimental and simulation results clearly show that the viscoelasticity of both the material and the contact surface contributes to compression creep, causing preload relaxation in composite bolted joints. As shown in Figure 14a, only the local region around the bolt/hole experiences compressive loading, highlighting the need to study compression creep behavior under localized loading.

This study conducted simulations to analyze compression creep under localized loading. As shown in Figure 14b, a 1/4 finite element model with symmetric boundary conditions was used for efficiency. Frictional contact was set between specimens 1 and 2, with an axial load applied to specimen 1, inducing localized loading on specimen 2. Displacement observations were made at reference points 1 and 2, with the bottom surface of specimen 2 fixed as a boundary condition.

**Figure 15:**
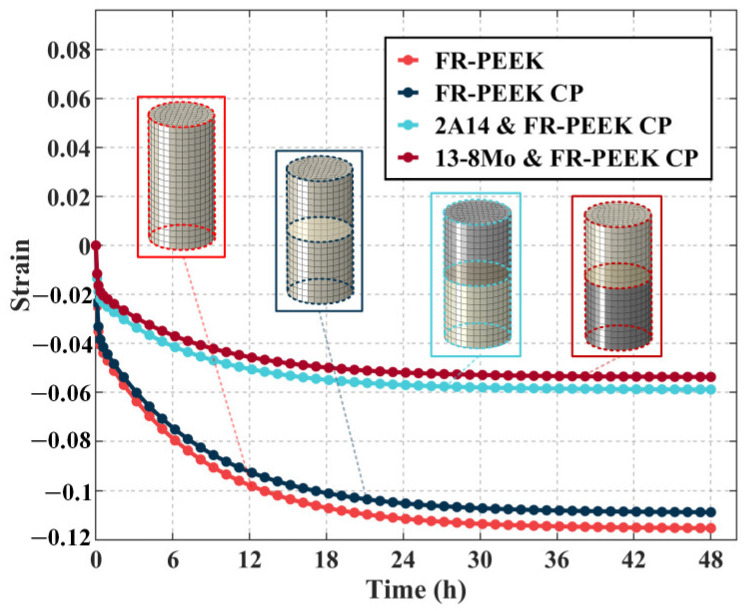
Comparison of the simulation results of FR-PEEK and contact pairs under localized loading.

Figure 15 presents the creep response of FR-PEEK and 2A14 under localized loading, compared with that of material specimens. Both materials exhibit a 28% reduction in strain under localized loading, suggesting that in composite/metal bolted joints, the contribution of bulk material creep to preload relaxation is reduced. It can be reasonably inferred that under a low initial load, creep at the rough contact surfaces exerts a greater influence on preload relaxation.

### 4.4. Structural Parameters

**Figure 16:**
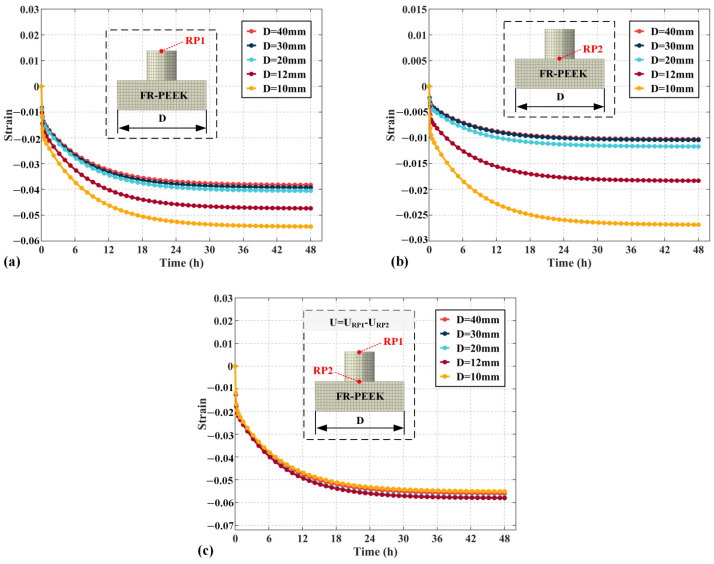
The influence of changes in edge length of specimens on creep strain under localized load: (**a**) contact pairs, (**b**) specimen 2, (**c**) specimen 1.

Figure 16a illustrates the influence of specimen side length D on the total strain, calculated from the displacement at reference point 1. As the side length increases, the strain decreases by 13% at 12 mm, 25% at 20 mm, 28% at 30 mm, and 30% at 40 mm. The results show that smaller specimen side lengths lead to increased strain, with the effect becoming more pronounced as the side length decreases. Figure 16b presents the strain of specimen 2, calculated through reference point 2. At increasing side lengths, the strain decreases by 32% at 12 mm, 56% at 20 mm, 61% at 30 mm, and 62% at 40 mm. The smaller the side length, the greater the impact on the creep behavior of the contact pair under localized loading. Figure 16c shows the strain of specimen 1, calculated through both reference points 1 and 2. The strain behavior of specimen 1 is largely unaffected by side length, suggesting that the side length primarily influences the strain of specimen 2, with minimal impact on specimen 1.

**Figure 17:**
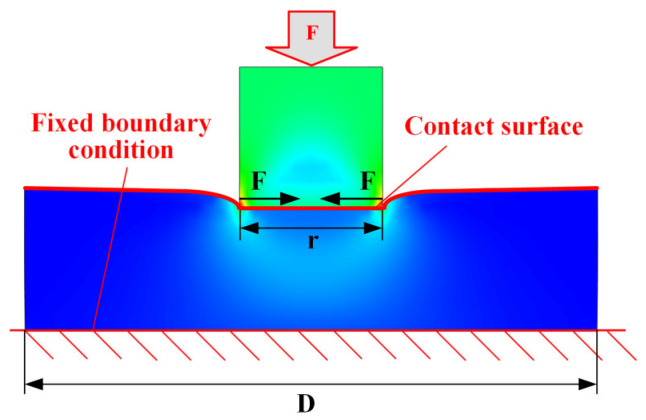
The contact pair under localized load (deformation amplified 30 times).

As shown in Figure 17, the localized loading acting on the surface of the material causes deformation in the surrounding regions, especially near the point of application. Simplifying this to a two-dimensional plane problem, the settlement displacement w at a point with distance x from the load can be expressed as:

(20) $$w=\frac{Q}{2\pi E}\left(\frac{1-\nu^{2}}{x}\right)$$

where *Q* represents the applied loading, N; *E* is the Young’s modulus of the material, MPa; ν is Poisson’s ratio; and x is the distance from the settlement point to the point of load application, mm. It can be observed that the closer the settlement point is to the load, the more significant the settlement becomes. The settlement displacement is inversely proportional to the distance. Therefore, as the dimensions of the two specimens, denoted by r and D, become increasingly similar, the material’s constraint effect diminishes, leading to a more pronounced increase in the compression creep strain due to the reduced resistance to deformation in the localized regions.

**Figure 18:**
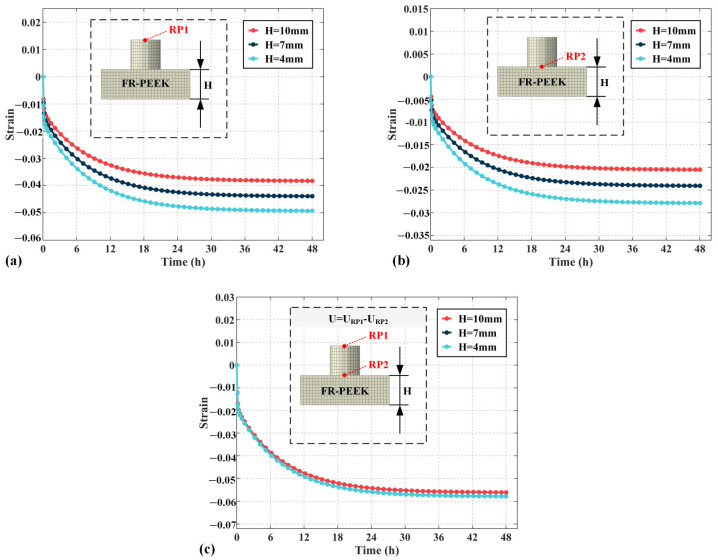
The influence of changes in the thickness of specimens on creep strain under localized load: (**a**) contact pairs, (**b**) specimen 2, (**c**) specimen 1.

Figure 18a shows the influence of specimen thickness *H* on strain, calculated from the displacement at reference point 1. When the thickness is reduced from 10 mm to 7 mm, the strain decreases by 11%, and at 4 mm, the decrease is 22%. The results indicate that as the thickness decreases, the strain increases linearly. Figure 18b presents the strain of specimen 2, calculated through reference point 2, which also increases linearly as the thickness decreases. Figure 18c shows the strain of specimen 1, calculated through both reference points 1 and 2, which is largely unaffected by thickness. Therefore, specimen thickness primarily affects the strain of specimen 2, with minimal impact on specimen 1.

**Figure 19:**
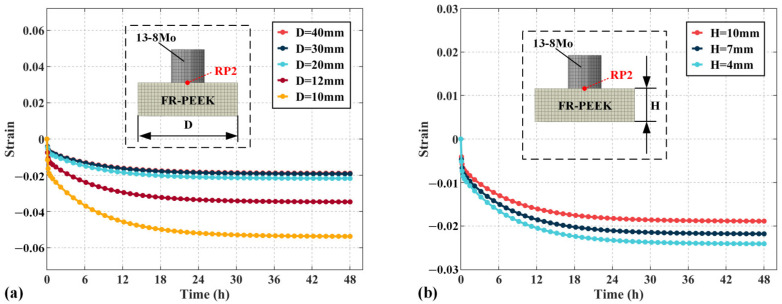
The influence of specimen dimension variations on creep strain under localized loading: (**a**) edge length, (**b**) thickness.

Figure 19 illustrates the compression behavior of heterogeneous materials under localized load, where specimen 1 represents 13-8Mo and specimen 2 represents FR-PEEK. Figure 19a shows the effect of the side length D of specimen 2 on strain. Since specimen 1 is elastic, only the behavior of specimen 2 is analyzed. As the side length increases, the strain decreases by 35% at 12 mm, 59% at 20 mm, 64% at 30 mm, and 65% at 40 mm. As the side length decreases, strain increases, with a greater impact at smaller side lengths. Unlike homogeneous contact pairs, the behavior of heterogeneous contact pairs is primarily determined by material 2, meaning strain is significantly influenced by the side length of this material. Figure 19b shows the effect of the thickness H of specimen 2 on strain. Reducing the thickness from 10 mm to 7 mm decreases the strain by 10% and by 22% at 4mm. These results demonstrate that as thickness decreases, strain increases linearly, similar to homogeneous contact pairs.

It can be observed that the strain of the specimen significantly decreases under localized loading. If the loaded area is sufficiently small, the strain seems to decrease by more than 60%. Therefore, for bolt joints, the connected component primarily experiences localized loading, and the contribution of the material’s creep is relatively small. As a result, the creep of rough contact surfaces may have a greater impact on the preload relaxation for composite bolted joints.

## 5. Conclusions

This paper presents a comprehensive study of the preload relaxation mechanism in composite bolted joints, combining experimental methods, theoretical analysis, and numerical simulations. Compression creep tests were conducted on materials and contact pairs to investigate the behavior of resin, FRP composites, metals, and both homogeneous and heterogeneous contact pairs. A non-stationary Burgers model was proposed to characterize the creep behavior of various materials. The model’s accuracy was validated through experiments and simulations. The main conclusions are as follows:

(1) A non-stationary Burgers model, based on a nonlinear viscoelastic correction term, was developed. The model parameters for PEEK, FR-PEEK, and 2A14 were fitted using nonlinear least squares, with results showing satisfactory performance compared to the classical Burgers model.
(2) A UMAT subroutine for the non-stationary Burgers model was developed in ABAQUS. Simulations using the fitted parameters showed that numerical results align with experimental data, confirming the subroutine’s correctness.
(3) Compression creep tests indicate that an increased initial load significantly enhances the structure’s resistance to creep. Moreover, the initial load exerts a considerable influence on the preload relaxation behavior of both FRP composites and metals.
(4) The strain was significantly smaller than under uniform loading under localized loading. If the loaded area is sufficiently small, strain can decrease by over 60%. Consequently, structural parameters significantly affect preload relaxation.

In conclusion, this study emphasizes the importance of initial load and structural parameters in preload relaxation and provides a reliable model and methodology for the design of composite bolted joints.

## Figures and Tables

**Table 1:** Properties of the materials examined.

Properties	Unit	Standard	PEEK	FR-PEEK
Tensile strength at yield	MPa	ISO 527 [48]	95	-
Elongation at yield	%	ISO 527	5	-
Tensile strength at break	MPa	ISO 527	-	113
Elongation at break	%	ISO 527	25	5
Modulus of elasticity in tension	MPa	ISO 527	4200	6300
Compression modulus	MPa	ISO 604 [49]	3400	-
Compression strength	MPa	ISO 604	23/43/102	29/52/120
Glass transition temperature	°C	ISO 11357 [50]	150	147
Heat distortion temperature HDT, Method A	°C	ISO-R 75 Method A [51]	162	-
Density	g/cm^3^	-	1.31	1.53

**Table 2:** Parameters for the classical and non-stationary Burgers models of materials.

Model	Material	*E*_1_/GPa	*η*_1_/GPa·h	*A*/GPa·h	*B*
The classical Burgers model	PEEK	54	13	2499	-
	FR-PEEK	66	17	2408	-
	2A14	630	56	290457	-
The non-stationary Burgers model	PEEK	71	6	329	0.321
	FR-PEEK	87	8	379	9.365
	2A14	3058	18	86	0.312

**Table 3:** Parameters for the classical and non-stationary Burgers models of contact pairs.

Model	Material	*E*_1_/GPa	*η*_1_/GPa·h	*A*/GPa·h	*B*	*R* ^2^
The classical Burgers model	PEEK	54	13	2499	-	0.8765
	FR-PEEK	66	17	2408	-	0.8846
	2A14	630	56	290,457	-	0.8252
The non-stationary Burgers model	PEEK	71	6	329	0.218	0.9679
	FR-PEEK	87	8	379	0.186	0.9803
	2A14	3058	18	86	9.087	0.8530

## Data Availability

All the results presented in the manuscript can be requested from the corresponding author.

## Abbreviations

FRP	Fiber-reinforced polymer
FEA	Finite element analysis
PEEK	Polyetheretherketone
FR-PEEK	Fiber-reinforced polyetheretherketone
UMAT	User-defined material subroutine
